# Neuron-Specific Enolase—What Are We Measuring?

**DOI:** 10.3390/ijms25095040

**Published:** 2024-05-06

**Authors:** Anastasiya S. Babkina, Maxim A. Lyubomudrov, Mikhail A. Golubev, Mikhail V. Pisarev, Arkady M. Golubev

**Affiliations:** 1 Federal Research and Clinical Center of Intensive Care Medicine and Rehabilitology, Moscow 107031, Russia; mlyubomudrov@fnkcrr.ru (M.A.L.); pisarev@gmail.com (M.V.P.); arkadygolubev@mail.ru (A.M.G.); 2 Medical Electronic Data Corporation, Moscow 119019, Russia; mik.golubev2013@yandex.ru

**Keywords:** NSE, biomarker, brain, ELISA, stroke

## Abstract

Since the discovery of the neuron-specific protein by Moore and McGregor in 1965, tens of thousands of studies have investigated the basic and applied significance of neuron-specific enolase (NSE). This promising biomarker, according to many researchers, has not found widespread use in clinical practice, particularly in acute cerebrovascular accidents. Moreover, the several studies refuting the usefulness of serum NSE measurement in critically ill patients leads us to consider the reasons for such contradictory conclusions. In this article, we have analyzed the main directions in the study of NSE and expressed our perspective on the reasons for the contradictory results and the difficulties in implementing the results of these studies in clinical practice. In our opinion, the method of the enzyme-linked immunosorbent assay (ELISA) used in the majority of the studies is inappropriate for the evaluation of NSE as a marker of central nervous system damage, because it does not allow for the differentiation of heterodimers of enolases and the assessment of the enzymatic activity of this group of enzymatic proteins. Therefore, the methodological approach for the evaluation of NSE (γγ-enolase) as a biomarker needs to be elaborated and improved. Furthermore, the specificity of the applied research methods and the appropriateness of the continued use of the term “neuron-specific enolase” must be addressed.

## 1. Introduction

Enolase was originally discovered in 1934 by Lohmann and Meyerhof while studying the conversion of 3-phosphoglycerate to pyruvate in muscle extracts [1]. Moore and McGregor identified the neuron-specific 14-3-2 protein in 1965 [2]. Because the protein exhibited enolase activity, it was later named neuron-specific enolase (NSE).

Enolases are essential for energy metabolism and participate in the glycolytic pathway, which converts glucose to pyruvate, produces ATP and NADH, and provides energy for cellular metabolism. Enolases are among the most abundant and highly expressed proteins in cells, from archaebacteria to mammals, with a highly conserved amino acid sequence [3]. The expression of the glycolytic enzyme enolase can vary depending on the energy requirements of the cells, as well as during development and in metabolic disorders caused by various factors [4].

The enolase isozymes in eukaryotes include enolase 1 (α), enolase 2 (γ), and enolase 3 (β), which are encoded by the *Eno1*, *Eno2*, and *Eno3* genes, respectively [5]. Furthermore, an enolase associated with sperm motility (ENOS/ENO4) has been identified in human and murine sperm [6]. The active form of enolase is dimeric. Isoforms form five different homodimers or heterodimers in cells (αα, αβ, αγ, ββ, γγ) [7].

The isozymes containing a γ subunit are known as the neuron-specific enolases (NSEs) [8]. NSE is widely distributed in central nervous system neurons [9]. It appears to be a marker for all neurons, neuroendocrine and paraneuronal cells [10]. The NSE levels vary between brain regions, ranging from 0.4% to 2.2% of the total soluble protein, with some neurons exhibiting NSE levels as high as 3–4% [11,12].

Since 1970, numerous scientific publications have been devoted to NSE research, as evidenced by the more than 13,000 articles retrieved by searching for “neuron-specific enolase” in the PubMed database and the 88,000 sources in the Google Scholar database. Despite significant interest, the results of NSE studies have not been widely translated into clinical practice. This raises the question of what factors contribute to the discrepancy between the intense interest in studying this molecular marker and the lack of practical application of NSE study results.

Many studies have shown that measuring NSE concentrations in biological fluids such as serum and cerebrospinal fluid (CSF) is clinically relevant [13,14,15,16]. Serum NSE levels are higher in patients with ischemic stroke than in healthy controls and correlate with infarct size and neurological deficits [17,18]. Several studies have demonstrated the prognostic significance of serum NSE concentrations in ischemic [17,19,20,21,22,23] and hemorrhagic stroke [24], hypertension [25,26], ischemia–reperfusion brain injury [27], and out-of-hospital cardiac arrest [28,29,30,31]. Based on these findings, the American Academy of Neurology recommended the use of serum NSE to predict adverse outcomes after global cerebral hypoperfusion in patients requiring cardiopulmonary resuscitation [32]. However, conflicting results of NSE studies and other objective circumstances have prevented the full implementation of these recommendations [31]. Thus, international guidelines suggest that the NSE level alone should not be used to predict poor neurological outcomes due to the high possibility of false positive results [33]. Moreover, the cutoff NSE level that is predictive of poor outcomes varies between studies [31].

NSE has also been actively studied in other diseases [4,34,35,36,37,38]. Increased serum NSE levels have been observed in lung diseases such as tuberculosis, chronic obstructive pulmonary disease, alveolar proteinosis, and acute respiratory distress syndrome [4]. In addition, patients with silicosis have been shown to display elevated serum NSE concentrations, which are helpful in diagnosing and assessing the severity of the disease [34]. Patients with severe respiratory failure caused by SARS-CoV-2 infection have been reported to have higher serum NSE levels than those with mild disease and controls [35]. Elevated serum NSE levels have been found in patients with small cell lung cancer [36]. NSE modulation regulates cell proliferation, drug resistance, and tumor growth [37]. NSE has been suggested as a potential biomarker for predicting the prognosis of gastric cancer [38].

Another group of studies found no clinical significance in measuring NSE [39,40,41,42,43,44,45]. Huţanu et al. found no significant differences in serum NSE concentrations between patients with ischemic stroke and controls, and high NSE levels were associated with a better outcome. Furthermore, NSE levels were not associated with functional outcomes. A study by Hutanu et al. questioned the use of NSE as a marker for ischemic stroke [39]. A systematic review by Anand et al. [40] found no link between NSE levels and functional outcomes or stroke severity. NSE has not been shown to help distinguish between ischemic and hemorrhagic stroke [41]. Conflicting results have been obtained regarding the validity of NSE concentrations in the late phase of ischemic stroke after endovascular treatment [42]. Pelinka et al. did not confirm the hypothesis that NSE is an early marker of traumatic brain injury (TBI) in multiple trauma. Serum NSE levels have been found to be elevated in patients with TBI to the same degree as in patients with multiple trauma without TBI [43]. A study to determine NSE levels to predict neurological outcome after cardiopulmonary resuscitation in a cohort of out-of-hospital cardiac arrest cases did not confirm previously proposed NSE thresholds based on the 2021 ERC guidelines [44]. There are no convincing data on the use of NSE in other areas of practical medicine (oncology and pediatrics). In particular, there is currently no evidence to support the use of serum NSE for the diagnosis and monitoring of neuroblastoma due to the high risk of false positive results associated with confounding factors (e.g., sample hemolysis) and other conditions (e.g., inflammation) [45].

Given the conflicting data on the clinical relevance of NSE, it is essential to analyze the key areas of study and determine the reasons for the contradictions and challenges in implementing the findings in clinical practice.

## 2. Characteristics of Brain Enolases

The study by Royds et al. [46] on the cellular localization of the enolase isoenzymes in the adult human brain showed that γγ enolase was present in the neurons and their axonal and dendritic processes, but not in the glial cells. Weak or negative staining was detected in ischemic neurons. The astrocytes, ependymal cells, capillary endothelial cells, Schwann cells, and arachnoid endothelium showed strong staining for αα enolase. No β-enolase was found in the brain cells. The authors of the study were unable to detect α-enolase in the neurons or γ-enolase in the non-neuronal cells in the adult human brain [46]. Therefore, αα enolase has been referred to as non-neuronal enolase (NNE) [12]. Immunohistochemical staining for γγ enolase has been observed in almost all types of neurons in the central and peripheral nervous system, except for specific neurons, such as cerebellar Purkinje cells [47]. Nevertheless, some studies have found weak positive staining of Purkinje cells at a particular stage of development [46,47,48,49,50]. In neurons and neuroendocrine cells, γ-enolase is present in the cytosol and associated with the synaptic plasma membrane [51]. Damaged axons were selectively labeled by immunohistochemical staining for NSE in diffuse axonal injury, whereas NSE was not detectable in intact axons [52].

According to Marangos et al., NSE (γγ enolase) is strictly localized to neurons, suggesting that the gene encoding the gamma subunit is expressed only in neuronal cells [53]. However, Deloulme et al. found γ-enolase transcripts in cultured neurons as well as in oligodendrocytes, astrocytes, and meningeal fibroblasts. The authors emphasized the need for caution when using neuron-specific enolase as a specific marker of neuronal cell differentiation [54]. The presence of non-neuronal enolase (NNE) in immature neurons and a shift from NNE to neuronal enolase (NSE) in mature neurons during neuronal differentiation have been reported [55]. Immunohistochemistry has shown that neurons stain positively for NNE during proliferation and migration and become positive for NSE only after they have settled in their final location, presumably after synaptic connections have been formed [47].

The primary functional difference between α-enolase and γ-enolase is their response to chloride ions, urea, and temperature [4]. α-enolase is more susceptible to these factors, whereas γ-enolase is more resistant to chloride-induced inactivation. γ-enolase exhibits significant resistance to chloride ions, which accumulate in neurons during repetitive depolarization. This resistance to chloride ions may have evolved to adapt to the intracellular environment of neurons and prevent the inactivation of chloride-sensitive enolase when metabolic energy is most needed [11].

The γ-subunits can dimerize with the α-subunit to form a heterodimer αγ enolase [4]. The gel electrophoresis of subunit profiles revealed bands for the α and γ subunits, suggesting the presence of a hybrid enolase variant in the brain [53]. According to Keller et al., the transcription of the α- and γ-enolase genes in different neurons across the brain regions indicates the formation of αγ hybrids in mature neurons. The gene expression of the α- and γ-enolase subunits varied among neuronal populations in the brain [56]. A study by Watanabe et al. [57] confirmed the expression of α- and γ-subunit mRNAs in adult brain neurons, resulting in similar temporal patterns throughout the brain, except for the cerebellum. The expression of the α-subunit in adult glial cells fell below the detection threshold of the in situ hybridization assay. These results suggest that both the α- and γ-enolase subunits are involved in energy production in mature brain neurons, and that the subunit composition of enolase varies depending on the neuron type and maturation [57].

Early studies identified NSE as γγ, but defined αγ as a hybrid form [47,50,53,58,59]. Recent studies have identified NSE as γγ and αγ dimers [9,12]. However, despite studies demonstrating the presence of a hybrid form in neurons, it is critical to determine whether this form is unique to neurons.

## 3. Hybrid αγ Enolase Is Commonly Found outside the Brain

Some pathological studies have questioned the specificity of NSE for neurons and neuroendocrine cells [60,61]. Studies showing NSE expression in tumors of non-neuroendocrine origin have cast doubt on the use of NSE as a diagnostic marker [62,63]. Therefore, pathologists often used the term “non-specific enolase” for this marker [64].

Studies showing that NSE is a non-specific marker are based on the presence of NSE outside the nervous and neuroendocrine systems [65,66]. Haimoto et al. showed the immunohistochemical localization of γ-enolase in smooth muscle cells of the aorta, prostate and uterine muscular and fibrotic tissue, myoepithelial cells, the cardiac conduction system, the epithelial cells of the loop of Henle, and the macula densa cells of the kidney [66].

Immunohistochemical staining for γ-enolase has also been demonstrated in spermatogonial cells, lymphocytes, plasma cells, platelets, and megakaryocytes, and, to a lesser ex-tent, in bronchial epithelial cells and type II alveolar epithelial cells of the lung and in the secretory cells of the fallopian tubes [66]. The distribution of αγ and γγ enolase was determined in various tissues using a sensitive enzyme-linked immunoassay system. Higher levels of αγ and γγ forms were found in the rectum, bladder, and uterus compared to other peripheral tissues [65]. Platelets and red blood cells contain mainly the αγ hybrid enolase combined with an αα isoenzyme [4,67,68].

One drawback is that the NSE detection antibody targeting the γγ form may cross-react with the αγ form of enolase [64,69]. This problem is present in both immunohistochemical and enzyme-linked immunosorbent assay (ELISA) methods and may result in non-specific binding. Anti-NSE serum reacts with both γγ and hybrid αγ enolases, rendering the specific radioimmunoassay ineffective [68]. Kato et al. found that neither assay system cross-reacted with the other homodimeric form of enolase. However, cross-reactivity was observed with a hybrid form of the enzyme that shares a common subunit with both homodimers [70]. The molecular form of the αγ enolase dimer may combine the properties of the γ and α subunits while having different properties to those of γγ and αα enolase [53,59].

The studies mentioned above have found that γγ and αγ enolase dimers are widely distributed outside the brain. Antibodies to the γ subunit, most commonly used to detect NSE, can detect both γγ enolase and a hybrid form of αγ enolase. Consequently, the conclusions of most studies are based on the identified “mixture” of γγ and αγ enolase dimers. This may lead to inaccurate conclusions regarding neuron-specific enolase levels because these molecular forms are not exclusive to neurons.

## 4. Methodology of the Enolase Study: What Are We Measuring?

The methodology of brain enolases studies should be guided by the following principles: (1) the determination of the subunit composition of enolase; (2) the separation of the γγ, αα, and hybrid αγ forms and measurement concentrations and activity corresponding to each molecular form of enolase; and (3) the evaluation of the cell specificity of the different enolase forms.

Biochemical methods such as chromatography and electrophoresis were used to isolate the molecular forms [71]. Column chromatography on diethylaminoethyl (DEAE) cellulose was used to isolate two isoenzymes of brain enolase (γγ and αα) and a hybrid molecular form of αγ enolase [72]. A quantitative serum NSE assay based on liquid chromatography–tandem mass spectrometry (LC-MS/MS) has been developed [73,74] and allows for the simultaneous determination of heterodimers by a characteristic peptide specific for the αγ subunit. To detect the hybrid form of αγ, solid-phase antibodies to one subunit and labeled complexes to the other can be used [70].

In the 1960s, a new method was proposed to detect endogenous plasma insulin using a radiolabeled antigen. This method was called radioimmunoassay (RIA) [75]. In the 1970s, an enzyme-linked immunosorbent assay (ELISA) was developed based on the principles of RIA by conjugating the target antigen (or antibody) with an enzyme instead of radioactive iodine-125 [76,77].

Eventually, ELISA became widely used and gradually replaced traditional biochemical methods. In our opinion, this replacement was a serious methodological error. Although ELISA is a highly sensitive method, its susceptibility to interference can lead to errors that result in erroneous conclusions and impact subsequent practical decisions [78]. First, conventional immunoassays are unable to discriminate between enolase isoenzymes [74]. Second, failure to measure enolase activity was another significant methodological error.

Specificity is required for the optimal use of biomarkers in medical practice [79]. Errors such as the measurement of the total γγ and γα enolase dimers, both of which contain the γ subunit, contribute to the lack of convincing data supporting the use of NSE for diagnostic and prognostic purposes. This method loses specificity due to the presence of αγ enolase in cells from different organs. The term “neuron-specific enolase” is misleading because γγ enolase and the hybrid molecular form of γα enolase have different structures, are produced by different cells, and may have different functions [53,59]. Many studies of neuron-specific enolase in ischemic and hemorrhagic stroke have relied on immunoenzymatic assays that detect both the γγ enolase isoenzyme and the hybrid form of γα enolase [39,40,41,42,43,44,45]. In this regard, the results of several studies on NSE in acute cerebrovascular accidents have been inconsistent.

## 5. Impact of Hemolysis on the Possibility of NSE Use in Clinical Practice

One of the major limitations of NSE quantification using ELISA is the occurrence of falsely elevated concentrations due to hemolysis [74], as the NSE isoform αγ is abundant in erythrocytes, and likely introduces errors [80]. Studies suggest that serum NSE originates from erythrocytes [74,80,81,82]. This is further compounded by the frequent practice of collecting blood samples from critically ill patients through indwelling intravenous catheters, where additional forces exerted on the erythrocytes result in more intense hemolysis [81]. The antibodies used in NSE assays specifically target the γ subunit only, but recognize both the γγ and αγ isoforms, thus being non-specific. This makes the enzyme-linked immunosorbent assay (ELISA) highly sensitive to the presence of red blood cell (RBC) hemolysis or platelet damage. Hemolysis accounts for 30–60% of biospecimen rejections in the preanalytical phase [82].

Several studies have confirmed that hemolysis, even latent, increases NSE concentration and can lead to erroneous results [74,81,82,83]. It has been reported that positive interference occurs when the concentration of cell-free hemoglobin in the serum is greater than 0.338 g/L and cannot be detected by visual inspection [84].

The correction of the NSE results in hemolyzed serum has been reported as a possible solution [85]. However, large inter-individual differences in erythrocyte NSE concentrations require the measurement of NSE in erythrocytes from a whole blood sample submitted simultaneously with a hemolyzed serum sample [81]. Unfortunately, this strategy seems difficult to effectively implement in clinical practice.

The NSE concentration in serum and CSF also depends on storage conditions such as temperature and time. CSF samples for NSE testing can be stored at −80 °C for a maximum of six months and serum samples for a maximum of nine months [83]. Because latent hemolysis increases NSE in serum samples, it is recommended to assess the intensity of hemolysis before deciding whether to measure NSE in a given sample [83].

Therefore, the dependence of the NSE concentration on hemolysis and the storage conditions significantly limits the use of NSE as a marker of neuronal damage. For example, a study by Motoyoshi et al. demonstrated the inappropriateness of NSE as a biomarker of brain damage immediately after cardiovascular surgery, because the results of neuron-specific enolase measurement are affected by hemolysis caused by cardiopulmonary bypass [86]. Separating homo- and heterodimers of NSE (γγ, αγ) and measuring the concentration of each molecular form of enolase could provide clarity to studies on the clinical significance of enolase.

## 6. Conclusions

The study of molecular forms of enolases is important in medicine, especially because glycolysis plays a critical role in organ and system metabolism during critical illness. However, a re-evaluation of methodological principles is a prerequisite for the successful development of this field of research.

Antibodies to the γ-subunit, commonly used to detect NSE, can recognize both the homodimers (γγ) and heterodimers (αγ) of enolase. The total concentration of the molecular forms of γγ and αγ enolase is the answer to the question “what are we detecting when we assay serum NSE?” This can lead to false conclusions about NSE because these molecular forms are not restricted to neurons but are widely distributed outside the brain. In addition, measuring the combined levels of these molecular forms provides an inaccurate estimate of the concentration of each form. The term “neuron-specific enolase” is misleading because both γγ enolase and its hybrid molecular form have different structures, localizations, and functions. As a result, the term “neuron-specific enolase” may not be entirely accurate, and it may be more appropriate to refer to enolase forms based on their subunit composition.

Methodological errors, such as measuring the total γγ and αγ enolase, both of which contain the γ subunit, result in contradictions and insufficient evidence to support the use of NSE for diagnostic and prognostic purposes. Distinguishing between the homodimers (γγ) and heterodimers (αγ) of enolase and measuring the concentrations of each molecular form may shed light on the clinical significance of these isoenzymes.

## Data Availability

Not applicable.
